# 1-[2-(2,4-Dinitro­benzyl­ideneamino)phen­yl]-3-phenyl­thio­urea

**DOI:** 10.1107/S1600536809035880

**Published:** 2009-09-12

**Authors:** M. Umadevi, S. Devaraj, M. Kandaswamy, G. Chakkaravarthi, V. Manivannan

**Affiliations:** a Department of Chemistry, Pallavan College of Engineering, Kanchipuram 631 502, Tamilnadu, India; b Deparment of Inorganic Chemistry, School of Chemical Sceinces, University of Madras, Guindy Campus, Chennai 600 025, India; c Department of Physics, CPCL Polytechnic College, Chennai 600 068, India; d Department of Research and Development, PRIST University, Vallam, Thanjavur 613 403, Tamilnadu, India

## Abstract

In the title compound, C_20_H_15_N_5_O_4_S, the central benzene ring makes dihedral angles of 59.5 (1) and 51.7 (1)°, respectively, with the terminal phenyl and benzene rings. The mol­ecular structure exhibits weak intra­molecular N—H⋯N and C—H⋯S inter­actions. In the crystal structure, mol­ecules are linked by weak inter­molecular N—H⋯S and C—H⋯O inter­actions, forming a chain along [1
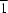
1].

## Related literature

For the biological activity of thio­ureas, see: Huebner *et al.* (1953 ▶); Madan & Taneja (1991 ▶); Manjula *et al.* (2009 ▶). For related structures, see: Gayathri *et al.* (2007 ▶, 2008 ▶). For graph-set notation, see: Bernstein *et al.* (1995 ▶).
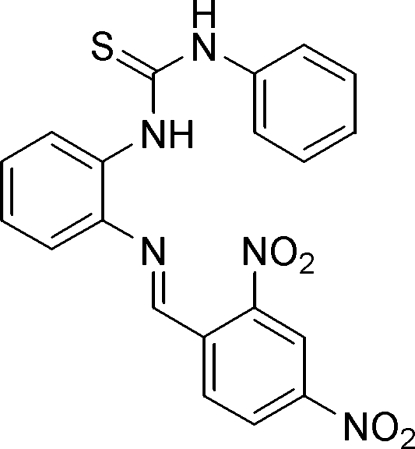


## Experimental

### 

#### Crystal data

C_20_H_15_N_5_O_4_S
*M* *_r_* = 421.43
Monoclinic, 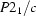

*a* = 8.362 (5) Å
*b* = 18.767 (3) Å
*c* = 12.379 (4) Å
β = 94.827 (5)°
*V* = 1935.7 (14) Å^3^
*Z* = 4
Mo *K*α radiation
μ = 0.21 mm^−1^
*T* = 295 K
0.20 × 0.16 × 0.16 mm

#### Data collection

Bruker Kappa APEXII diffractometer
Absorption correction: multi-scan (**SADABS**; Sheldrick, 1996 ▶) *T*_min_ = 0.960, *T*_max_ = 0.968
28336 measured reflections
6878 independent reflections
4509 reflections with *I* > 2σ(*I*)
*R*_int_ = 0.028

#### Refinement

*R*[*F* ^2^ > 2σ(*F* ^2^)] = 0.046
*wR*(*F* ^2^) = 0.137
*S* = 1.02
6878 reflections
271 parameters
H-atom parameters constrained
Δρ_max_ = 0.25 e Å^−3^
Δρ_min_ = −0.37 e Å^−3^

### 

Data collection: *APEX2* (Bruker, 2004 ▶); cell refinement: *SAINT* (Bruker, 2004 ▶); data reduction: *SAINT*; program(s) used to solve structure: *SHELXS97* (Sheldrick, 2008 ▶); program(s) used to refine structure: *SHELXL97* (Sheldrick, 2008 ▶); molecular graphics: *PLATON* (Spek, 2009 ▶); software used to prepare material for publication: *SHELXL97*.

## Supplementary Material

Crystal structure: contains datablocks global, I. DOI: 10.1107/S1600536809035880/is2457sup1.cif

Structure factors: contains datablocks I. DOI: 10.1107/S1600536809035880/is2457Isup2.hkl

Additional supplementary materials:  crystallographic information; 3D view; checkCIF report

## Figures and Tables

**Table 1:** Hydrogen-bond geometry (Å, °)

*D*—H⋯*A*	*D*—H	H⋯*A*	*D*⋯*A*	*D*—H⋯*A*
N4—H4*A*⋯N3	0.86	2.14	2.614 (2)	114
C3—H3⋯S1	0.93	2.55	3.215 (2)	128
N4—H4*A*⋯N3	0.86	2.14	2.614 (2)	114
N5—H5*A*⋯S1^i^	0.86	2.49	3.284 (2)	155
C12—H12⋯O3^ii^	0.93	2.57	3.397 (3)	148

Symmetry codes: (i) 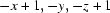; (ii) 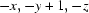.

**Fig. 1.**
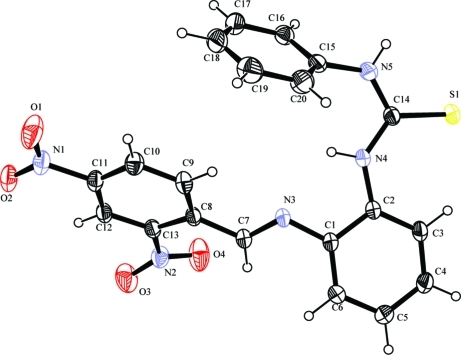
The molecular structure of (I), with atom labels and 30% probability displacement ellipsoids for non-H atoms.

**Fig. 2.**
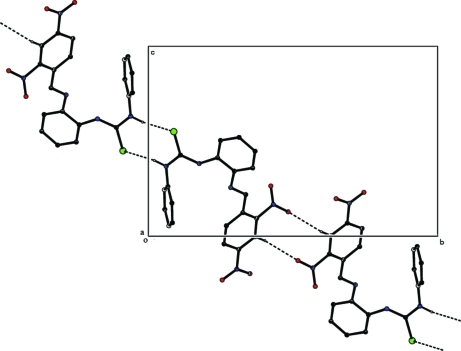
The packing of (I), viewed down the a axis. Hydrogen bonds are shown as dashed lines. H atoms not involved in hydrogen bonding have been omitted.

## supplementary crystallographic information

### Comment

Thioureas are known to exhibit antiviral, antibacterial, anticancer (Madan & Taneja, 1991; Manjula *et al.*, 2009), antifungal, antitubercular, antithyroidal, herbicidal and insecticidal (Huebner *et al.*, 1953) activities.

The geometric parameters in (I), (Fig. 1) agree with the reported values of similar structures (Gayathri *et al.*, 2007, 2008). The benzene ring C1—C6 makes the dihedral angle of 59.5 (1)° with the phenyl ring C15—C20 and 51.7 (1)° with the dinitrobenzene ring C8—C13.

The molecular structure of (I) exhibits weak intramolecular N—H···N, C—H···S and C—H···O interactions and the crystal structure is stabilized by weak intermolecular N—H···S and C—H···O interactions (Table 1 and Fig. 2). The intermolecular N5—H5A···S1 interaction generates an eight-membered ring, with graph-set motif *R*_2_^2^(8) and the C12—H12···O3 interaction generates a ten-membered ring, with graph-set motif *R*_2_^2^(10) (Bernstein, 1995).

### Experimental

To the solution of 1-(2-aminophenyl)-3-phenylthiourea (0.3 g, 1.2 mmol) in methanol (25 ml), 2,4-dinitrobenzaldehyde (0.36 g, 1.2 mmol) in methanol (25 ml) was added under stirring. The resulting mixture was refluxed for 3 h and cooled to room temperature. The solid product was collected by filtration and washed with cold methanol. The microcrystalline compound was recrystallized from hot chloroform.

### Refinement

H atoms were positioned geometrically and refined using riding model, with C—H = 0.93 Å and *U*_iso_(H) = 1.2*U*_eq_(C), and with N—H = 0.86 Å and *U*_iso_(H) = 1.2*U*_eq_(N).

### Crystal data

**Table:**

C_20_H_15_N_5_O_4_S	*F*(000) = 872
*M**_r_* = 421.43	*D*_x_ = 1.446 Mg m^−^^3^
Monoclinic, *P*2_1_/*c*	Mo *K*α radiation, λ = 0.71073 Å
Hall symbol: -P 2ybc	Cell parameters from 9702 reflections
*a* = 8.362 (5) Å	θ = 2.2–32.3°
*b* = 18.767 (3) Å	µ = 0.21 mm^−^^1^
*c* = 12.379 (4) Å	*T* = 295 K
β = 94.827 (5)°	Prism, orange
*V* = 1935.7 (14) Å^3^	0.20 × 0.16 × 0.16 mm
*Z* = 4	

### Data collection

**Table:**

Bruker Kappa APEXII diffractometer	6878 independent reflections
Radiation source: fine-focus sealed tube	4509 reflections with *I* > 2σ(*I*)
graphite	*R*_int_ = 0.028
ω and φ scans	θ_max_ = 32.3°, θ_min_ = 2.2°
Absorption correction: multi-scan (*SADABS*; Sheldrick, 1996)	*h* = −12→12
*T*_min_ = 0.960, *T*_max_ = 0.968	*k* = −28→26
28336 measured reflections	*l* = −9→18

### Refinement

**Table:**

Refinement on *F*^2^	Primary atom site location: structure-invariant direct methods
Least-squares matrix: full	Secondary atom site location: difference Fourier map
*R*[*F*^2^ > 2σ(*F*^2^)] = 0.046	Hydrogen site location: inferred from neighbouring sites
*wR*(*F*^2^) = 0.137	H-atom parameters constrained
*S* = 1.02	*w* = 1/[σ^2^(*F*_o_^2^) + (0.0628*P*)^2^ + 0.3472*P*] where *P* = (*F*_o_^2^ + 2*F*_c_^2^)/3
6878 reflections	(Δ/σ)_max_ < 0.001
271 parameters	Δρ_max_ = 0.25 e Å^−^^3^
0 restraints	Δρ_min_ = −0.37 e Å^−^^3^

### Fractional atomic coordinates and isotropic or equivalent isotropic displacement parameters (Å^2^)

**Table:**

	*x*	*y*	*z*	*U*_iso_*/*U*_eq_	
C1	0.62398 (16)	0.29698 (7)	0.34283 (10)	0.0381 (3)	
C2	0.65955 (15)	0.23913 (7)	0.41328 (9)	0.0360 (3)	
C3	0.76661 (18)	0.24941 (8)	0.50420 (11)	0.0448 (3)	
H3	0.7860	0.2127	0.5541	0.054*	
C4	0.84381 (19)	0.31360 (9)	0.52044 (12)	0.0511 (4)	
H4	0.9149	0.3200	0.5816	0.061*	
C5	0.8174 (2)	0.36867 (9)	0.44751 (13)	0.0560 (4)	
H5	0.8740	0.4111	0.4576	0.067*	
C6	0.70627 (19)	0.36049 (8)	0.35919 (12)	0.0496 (4)	
H6	0.6868	0.3979	0.3106	0.060*	
C7	0.42126 (16)	0.33645 (8)	0.21914 (10)	0.0412 (3)	
H7	0.4321	0.3809	0.2522	0.049*	
C8	0.30541 (16)	0.32650 (7)	0.12401 (10)	0.0381 (3)	
C9	0.32496 (18)	0.26958 (8)	0.05491 (11)	0.0459 (3)	
H9	0.4040	0.2359	0.0739	0.055*	
C10	0.23061 (19)	0.26145 (9)	−0.04113 (11)	0.0478 (3)	
H10	0.2441	0.2225	−0.0859	0.057*	
C11	0.11616 (16)	0.31229 (8)	−0.06909 (10)	0.0425 (3)	
C12	0.08777 (17)	0.36881 (8)	−0.00335 (11)	0.0424 (3)	
H12	0.0087	0.4023	−0.0234	0.051*	
C13	0.18123 (16)	0.37421 (8)	0.09399 (10)	0.0393 (3)	
C14	0.57689 (16)	0.11081 (7)	0.42739 (10)	0.0387 (3)	
C15	0.41735 (19)	0.06746 (7)	0.26115 (11)	0.0435 (3)	
C16	0.2548 (2)	0.05512 (8)	0.24467 (12)	0.0471 (3)	
H16	0.1955	0.0457	0.3034	0.057*	
C17	0.1798 (2)	0.05672 (10)	0.14094 (14)	0.0606 (4)	
H17	0.0700	0.0482	0.1298	0.073*	
C18	0.2668 (3)	0.07077 (10)	0.05446 (13)	0.0687 (5)	
H18	0.2161	0.0721	−0.0153	0.082*	
C19	0.4278 (3)	0.08286 (11)	0.07051 (14)	0.0703 (5)	
H19	0.4862	0.0923	0.0114	0.084*	
C20	0.5058 (2)	0.08133 (10)	0.17423 (13)	0.0599 (4)	
H20	0.6157	0.0895	0.1849	0.072*	
N1	0.01932 (16)	0.30558 (9)	−0.17349 (10)	0.0534 (3)	
N2	0.14010 (17)	0.43172 (8)	0.16707 (11)	0.0542 (3)	
N3	0.50634 (14)	0.28461 (6)	0.25608 (9)	0.0406 (3)	
N4	0.58017 (14)	0.17574 (6)	0.38222 (8)	0.0409 (3)	
H4A	0.5216	0.1791	0.3219	0.049*	
N5	0.49272 (17)	0.06127 (7)	0.36821 (10)	0.0529 (3)	
H5A	0.4832	0.0206	0.3990	0.064*	
O1	0.0455 (2)	0.25501 (10)	−0.23077 (11)	0.0917 (5)	
O2	−0.08088 (15)	0.35115 (8)	−0.19739 (9)	0.0652 (3)	
O3	0.0704 (2)	0.48301 (8)	0.12851 (13)	0.0930 (5)	
O4	0.1705 (2)	0.42321 (9)	0.26429 (11)	0.0902 (5)	
S1	0.66368 (5)	0.08789 (2)	0.55035 (3)	0.04838 (12)	

### Atomic displacement parameters (Å^2^)

**Table:**

	*U*^11^	*U*^22^	*U*^33^	*U*^12^	*U*^13^	*U*^23^
C1	0.0389 (7)	0.0402 (7)	0.0341 (5)	0.0022 (5)	−0.0038 (5)	0.0060 (5)
C2	0.0366 (7)	0.0377 (7)	0.0330 (5)	0.0040 (5)	−0.0017 (5)	0.0047 (5)
C3	0.0487 (8)	0.0449 (8)	0.0384 (6)	0.0049 (6)	−0.0100 (5)	0.0062 (5)
C4	0.0529 (9)	0.0506 (9)	0.0465 (7)	0.0005 (7)	−0.0159 (6)	−0.0013 (6)
C5	0.0595 (10)	0.0439 (8)	0.0609 (9)	−0.0082 (7)	−0.0171 (7)	0.0021 (7)
C6	0.0544 (9)	0.0403 (8)	0.0516 (8)	−0.0045 (6)	−0.0108 (6)	0.0107 (6)
C7	0.0429 (7)	0.0426 (7)	0.0368 (6)	0.0018 (6)	−0.0041 (5)	0.0065 (5)
C8	0.0371 (7)	0.0428 (7)	0.0335 (5)	0.0017 (5)	−0.0012 (5)	0.0091 (5)
C9	0.0442 (8)	0.0509 (8)	0.0416 (6)	0.0108 (6)	−0.0018 (5)	0.0056 (6)
C10	0.0498 (8)	0.0550 (9)	0.0381 (6)	0.0062 (7)	0.0015 (6)	−0.0022 (6)
C11	0.0374 (7)	0.0572 (9)	0.0322 (5)	−0.0021 (6)	−0.0015 (5)	0.0077 (5)
C12	0.0375 (7)	0.0482 (8)	0.0405 (6)	0.0042 (6)	−0.0030 (5)	0.0106 (5)
C13	0.0403 (7)	0.0408 (7)	0.0364 (6)	0.0024 (6)	0.0002 (5)	0.0062 (5)
C14	0.0401 (7)	0.0392 (7)	0.0364 (6)	0.0017 (5)	−0.0001 (5)	0.0072 (5)
C15	0.0586 (9)	0.0340 (7)	0.0369 (6)	−0.0010 (6)	−0.0032 (6)	0.0032 (5)
C16	0.0558 (9)	0.0397 (7)	0.0455 (7)	0.0040 (7)	0.0019 (6)	−0.0005 (6)
C17	0.0665 (11)	0.0515 (10)	0.0602 (9)	0.0048 (8)	−0.0163 (8)	−0.0025 (7)
C18	0.1052 (16)	0.0567 (11)	0.0405 (7)	0.0006 (10)	−0.0150 (8)	−0.0024 (7)
C19	0.1034 (17)	0.0702 (12)	0.0388 (7)	−0.0056 (11)	0.0143 (9)	0.0021 (7)
C20	0.0657 (11)	0.0657 (11)	0.0489 (8)	−0.0085 (9)	0.0079 (7)	0.0050 (7)
N1	0.0498 (7)	0.0732 (10)	0.0357 (5)	−0.0006 (7)	−0.0040 (5)	0.0038 (6)
N2	0.0549 (8)	0.0525 (8)	0.0531 (7)	0.0126 (6)	−0.0072 (6)	−0.0041 (6)
N3	0.0413 (6)	0.0429 (6)	0.0360 (5)	−0.0013 (5)	−0.0064 (4)	0.0103 (4)
N4	0.0464 (6)	0.0384 (6)	0.0358 (5)	−0.0013 (5)	−0.0096 (4)	0.0074 (4)
N5	0.0704 (9)	0.0438 (7)	0.0420 (6)	−0.0131 (6)	−0.0106 (6)	0.0141 (5)
O1	0.1080 (12)	0.1083 (13)	0.0534 (7)	0.0283 (10)	−0.0248 (7)	−0.0255 (8)
O2	0.0583 (7)	0.0850 (9)	0.0490 (6)	0.0074 (6)	−0.0157 (5)	0.0116 (6)
O3	0.1231 (13)	0.0625 (9)	0.0886 (10)	0.0423 (9)	−0.0200 (9)	−0.0069 (7)
O4	0.1129 (13)	0.1065 (12)	0.0489 (7)	0.0467 (10)	−0.0073 (7)	−0.0162 (7)
S1	0.0547 (2)	0.0481 (2)	0.04016 (17)	−0.00134 (16)	−0.00860 (14)	0.01530 (14)

### Geometric parameters (Å, °)

**Table:**

C1—C6	1.383 (2)	C12—H12	0.9300
C1—C2	1.4085 (17)	C13—N2	1.468 (2)
C1—N3	1.4132 (16)	C14—N4	1.3419 (17)
C2—C3	1.3914 (18)	C14—N5	1.3452 (19)
C2—N4	1.4003 (17)	C14—S1	1.6860 (13)
C3—C4	1.374 (2)	C15—C16	1.377 (2)
C3—H3	0.9300	C15—C20	1.381 (2)
C4—C5	1.378 (2)	C15—N5	1.4236 (17)
C4—H4	0.9300	C16—C17	1.381 (2)
C5—C6	1.383 (2)	C16—H16	0.9300
C5—H5	0.9300	C17—C18	1.370 (3)
C6—H6	0.9300	C17—H17	0.9300
C7—N3	1.2678 (17)	C18—C19	1.363 (3)
C7—C8	1.4725 (18)	C18—H18	0.9300
C7—H7	0.9300	C19—C20	1.391 (2)
C8—C9	1.387 (2)	C19—H19	0.9300
C8—C13	1.3975 (19)	C20—H20	0.9300
C9—C10	1.379 (2)	N1—O1	1.215 (2)
C9—H9	0.9300	N1—O2	1.2162 (19)
C10—C11	1.375 (2)	N2—O3	1.2031 (18)
C10—H10	0.9300	N2—O4	1.2194 (18)
C11—C12	1.370 (2)	N4—H4A	0.8600
C11—N1	1.4716 (17)	N5—H5A	0.8600
C12—C13	1.3837 (18)		
			
C6—C1—C2	119.82 (12)	C12—C13—N2	116.50 (12)
C6—C1—N3	123.98 (12)	C8—C13—N2	120.97 (12)
C2—C1—N3	116.18 (12)	N4—C14—N5	115.39 (11)
C3—C2—N4	126.71 (12)	N4—C14—S1	125.83 (11)
C3—C2—C1	118.81 (13)	N5—C14—S1	118.76 (10)
N4—C2—C1	114.48 (11)	C16—C15—C20	120.20 (14)
C4—C3—C2	120.16 (13)	C16—C15—N5	118.58 (13)
C4—C3—H3	119.9	C20—C15—N5	121.10 (15)
C2—C3—H3	119.9	C15—C16—C17	119.97 (15)
C3—C4—C5	120.97 (13)	C15—C16—H16	120.0
C3—C4—H4	119.5	C17—C16—H16	120.0
C5—C4—H4	119.5	C18—C17—C16	120.07 (18)
C4—C5—C6	119.67 (15)	C18—C17—H17	120.0
C4—C5—H5	120.2	C16—C17—H17	120.0
C6—C5—H5	120.2	C19—C18—C17	120.09 (15)
C5—C6—C1	120.30 (13)	C19—C18—H18	120.0
C5—C6—H6	119.9	C17—C18—H18	120.0
C1—C6—H6	119.9	C18—C19—C20	120.80 (17)
N3—C7—C8	120.17 (13)	C18—C19—H19	119.6
N3—C7—H7	119.9	C20—C19—H19	119.6
C8—C7—H7	119.9	C15—C20—C19	118.87 (18)
C9—C8—C13	116.94 (12)	C15—C20—H20	120.6
C9—C8—C7	119.16 (12)	C19—C20—H20	120.6
C13—C8—C7	123.75 (13)	O1—N1—O2	124.14 (13)
C10—C9—C8	121.91 (13)	O1—N1—C11	117.79 (14)
C10—C9—H9	119.0	O2—N1—C11	118.06 (14)
C8—C9—H9	119.0	O3—N2—O4	123.37 (15)
C11—C10—C9	118.42 (14)	O3—N2—C13	118.34 (13)
C11—C10—H10	120.8	O4—N2—C13	118.15 (13)
C9—C10—H10	120.8	C7—N3—C1	118.79 (12)
C12—C11—C10	122.65 (12)	C14—N4—C2	133.07 (11)
C12—C11—N1	118.55 (13)	C14—N4—H4A	113.5
C10—C11—N1	118.80 (14)	C2—N4—H4A	113.5
C11—C12—C13	117.43 (13)	C14—N5—C15	128.38 (12)
C11—C12—H12	121.3	C14—N5—H5A	115.8
C13—C12—H12	121.3	C15—N5—H5A	115.8
C12—C13—C8	122.50 (13)		
			
C6—C1—C2—C3	6.0 (2)	N5—C15—C16—C17	−176.32 (14)
N3—C1—C2—C3	−175.84 (12)	C15—C16—C17—C18	−0.2 (2)
C6—C1—C2—N4	−173.89 (14)	C16—C17—C18—C19	0.3 (3)
N3—C1—C2—N4	4.25 (18)	C17—C18—C19—C20	−0.2 (3)
N4—C2—C3—C4	175.60 (15)	C16—C15—C20—C19	0.3 (3)
C1—C2—C3—C4	−4.3 (2)	N5—C15—C20—C19	176.38 (16)
C2—C3—C4—C5	−0.2 (3)	C18—C19—C20—C15	−0.1 (3)
C3—C4—C5—C6	3.0 (3)	C12—C11—N1—O1	−179.74 (16)
C4—C5—C6—C1	−1.2 (3)	C10—C11—N1—O1	−0.1 (2)
C2—C1—C6—C5	−3.3 (2)	C12—C11—N1—O2	0.8 (2)
N3—C1—C6—C5	178.69 (15)	C10—C11—N1—O2	−179.62 (15)
N3—C7—C8—C9	−20.9 (2)	C12—C13—N2—O3	−24.3 (2)
N3—C7—C8—C13	163.79 (13)	C8—C13—N2—O3	157.70 (17)
C13—C8—C9—C10	2.3 (2)	C12—C13—N2—O4	151.72 (17)
C7—C8—C9—C10	−173.30 (14)	C8—C13—N2—O4	−26.3 (2)
C8—C9—C10—C11	1.1 (2)	C8—C7—N3—C1	175.78 (12)
C9—C10—C11—C12	−2.8 (2)	C6—C1—N3—C7	−31.3 (2)
C9—C10—C11—N1	177.57 (13)	C2—C1—N3—C7	150.61 (13)
C10—C11—C12—C13	0.9 (2)	N5—C14—N4—C2	−175.70 (14)
N1—C11—C12—C13	−179.52 (12)	S1—C14—N4—C2	5.9 (2)
C11—C12—C13—C8	2.9 (2)	C3—C2—N4—C14	2.5 (2)
C11—C12—C13—N2	−175.14 (13)	C1—C2—N4—C14	−177.57 (14)
C9—C8—C13—C12	−4.4 (2)	N4—C14—N5—C15	5.2 (2)
C7—C8—C13—C12	171.00 (13)	S1—C14—N5—C15	−176.34 (13)
C9—C8—C13—N2	173.51 (13)	C16—C15—N5—C14	−121.98 (17)
C7—C8—C13—N2	−11.1 (2)	C20—C15—N5—C14	61.8 (2)
C20—C15—C16—C17	−0.1 (2)		

### Hydrogen-bond geometry (Å, °)

**Table:**

*D*—H···*A*	*D*—H	H···*A*	*D*···*A*	*D*—H···*A*
N4—H4A···N3	0.86	2.14	2.614 (2)	114
C3—H3···S1	0.93	2.55	3.215 (2)	128
C7—H7···O4	0.93	2.34	2.749 (3)	106
N4—H4A···N3	0.86	2.14	2.614 (2)	114
N5—H5A···S1^i^	0.86	2.49	3.284 (2)	155
C12—H12···O3^ii^	0.93	2.57	3.397 (3)	148

Symmetry codes: (i) −*x*+1, −*y*, −*z*+1; (ii) −*x*, −*y*+1, −*z*.
